# Transgenic disruption of endogenous glucocorticoid signaling in osteoblasts does not alter long-term K/BxN serum transfer-induced arthritis

**DOI:** 10.1186/s13075-023-03112-9

**Published:** 2023-08-04

**Authors:** Tazio Maleitzke, Edgar Wiebe, Dörte Huscher, Cornelia M. Spies, Jinwen Tu, Timo Gaber, Yu Zheng, Frank Buttgereit, Markus J. Seibel, Hong Zhou

**Affiliations:** 1 Bone Research Program, ANZAC Research Institute, University of Sydney, Sydney, NSW Australia; 2 Center for Musculoskeletal Surgery, Charité – Universitätsmedizin Berlin, corporate member of Freie Universität Berlin and Humboldt-Universität Zu Berlin, Berlin, Germany; 3 Julius Wolff Institute, Berlin Institute of Health at Charité – Universitätsmedizin Berlin, Berlin, Germany; 4 BIH Charité Clinician Scientist Program, BIH Biomedical Innovation Academy, Berlin Institute of Health at Charité – Universitätsmedizin Berlin, Berlin, Germany; 5 Department of Rheumatology and Clinical Immunology, Charité – Universitätsmedizin Berlin, corporate member of Freie Universität Berlin and Humboldt-Universität Zu Berlin, Berlin, Germany; 6 Institute of Biometry and Clinical Epidemiology, Charité – Universitätsmedizin Berlin, corporate member of Freie Universität Berlin and Humboldt-Universität Zu Berlin, Berlin Institute of Health, Berlin, Germany; 7 Department of Endocrinology and Metabolism, Concord Repatriation Hospital, University of Sydney, Sydney, NSW Australia

**Keywords:** 11ß-HSD2, Cortisol, Cortisone, Rheumatoid arthritis, Antibody, Joint inflammation, Cartilage, Bone

## Abstract

**Background:**

Disruption of glucocorticoid (GC) signaling in osteoblasts results in a marked attenuation of acute antibody-induced arthritis. The role of endogenous GCs in chronic inflammatory arthritis is however not fully understood. Here, we investigated the impact of endogenous GC signaling in osteoblasts on inflammation and bone integrity under chronic inflammatory arthritis by inactivating osteoblastic GC signaling in a long-term K/BxN serum transfer-induced induced arthritis (STIA) model.

**Methods:**

Intracellular GC signaling in osteoblasts was disrupted by transgenic (tg) overexpression of 11beta-hydroxysteroid dehydrogenase type 2 (11ß-HSD2). Inflammatory arthritis was induced in 5-week-old male tg mice and their wild type (WT) littermates by intraperitoneal (i.p.) injection of K/BxN serum while controls (CTRLs) received phosphate-buffered saline (PBS). In a first cohort, K/BxN STIA was allowed to abate until  the endpoint of 42 days (STIA). To mimic rheumatic flares, a second cohort was additionally injected on days 14 and 28 with K/BxN serum (STIA ^boost^). Arthritis severity was assessed daily by clinical scoring and ankle size measurements. Ankle joints were assessed histopathologically. Systemic effects of inflammation on long bone metabolism were analyzed in proximal tibiae by micro-computed tomography (μCT) and histomorphometry.

**Results:**

Acute arthritis developed in both tg and WT mice (STIA and STIA ^boost^) and peaked around day 8. While WT STIA and tg STIA mice showed a steady decline of inflammation until day 42, WT STIA ^boost^ and tg STIA ^boost^ mice exhibited an arthritic phenotype over a period of 42 days. Clinical arthritis severity did not differ significantly between WT and tg mice, neither in the STIA nor in the STIA ^boost^ cohorts. Correspondingly, histological indices of inflammation, cartilage damage, and bone erosion showed no significant difference between WT and tg mice on day 42. Histomorphometry revealed an increased bone turnover in tg CTRL and tg STIA ^boost^ compared to WT CTRL and WT STIA ^boost^ animals, respectively.

**Conclusions:**

In contrast to the previously reported modulating effects of endogenous GC signaling in osteoblasts during acute K/BxN STIA, this effect seems to perish during the chronic inflammatory and resolution phase. These findings indicate that endogenous GC signaling in osteoblasts may mainly be relevant during acute and subacute inflammatory processes.

## Background

Due to their potent anti-inflammatory and immunomodulatory properties, glucocorticoids (GCs) play an important therapeutic role in controlling inflammatory autoimmune disease activity [1, 2].

Although their pharmacological actions and clinical (side-) effects are well characterized, the role of endogenous GCs in autoimmune arthritis is less well-understood [3, 4]. We previously showed that endogenous GCs are indispensable for osteoblastic differentiation and skeletal maturation through a Wnt-dependent pathway [5, 6].

In rheumatoid arthritis (RA), chronic inflammation leads to structural bone damage locally (in the form of bone erosions) and systemically (in the form of osteoporosis), with osteoblasts representing key immunocompetent players in bone turnover regulation [7].

In response to inflammatory stimuli, the expression of 11ß-hydroxysteroid dehydrogenase type 1 (11β-HSD1), which converts inactive endogenous cortisone to active cortisol, is upregulated in both osteoblasts and synoviocytes [8, 9], and high RA disease activity is associated with increased levels of 11β-HSD1 in the synovium [10].

We previously studied the effect of transgenic (tg) disruption of endogenous GC signaling through an overexpression of 11ß-HSD2 (which converts cortisol to cortisone) in mature osteoblasts and osteocytes in murine antibody-mediated arthritis. We showed that the inactivation of GC signaling in osteoblasts attenuated T-cell-independent K/BxN serum transfer-induced (STIA) and collagen II antibody-induced arthritis (CAIA) in the acute and subacute phases following disease induction [11, 12]. Further studies by our group showed that the T-cell-dependent model of antigen-induced arthritis (AIA) was however not affected by disrupted osteoblastic GC signaling. This suggested that the mechanisms by which osteoblasts and osteocytes are able to regulate inflammation involve the innate rather than the adaptive immune system via a GC-dependent pathway [13].

In our previous work, the effect of endogenous GC disruption in osteoblasts on murine K/BxN STIA was only analyzed during the acute and subacute phases of day 0 to 14. As expected, the observed degree of bone erosions and systemic bone loss was, overall, rather mild [11].

However, RA is a chronic inflammatory disease, where morbidity and bone destruction increase over time [14, 15]. For experimental murine models of arthritis, data on chronic inflammatory changes are scarce, and the effects of chronic inflammation and arthritis resolution on bone are not well-studied.

In 2012, Matzelle et al. conducted a long-term experiment employing the K/BxN STIA model in C57BL/6 WT mice, to study the chronic and resolution phases of arthritis. As clinical inflammation regressed around day 15, RANKL expression, and osteoclast numbers decreased and anabolic and pro-matrix mineralization factors, including osteoprotegerin (OPG), increased [16]. While the study characterized the chronic resolution phase of antibody-mediated arthritis in WT animals, the effects of osteoblastic GC signaling in chronic experimental arthritis remained unclear.

Thus, we investigated whether transgenically disrupted GC signaling in osteoblasts impacts inflammation and bone in (a) the resolution phase, where arthritis gradually abates due to its natural and transient course (STIA), and (b) chronically active K/BxN STIA maintained by repeated boost-injections of the arthritogenic serum (STIA ^boost^).

## Materials and methods

### Transgenic mouse model

Disruption of GC signaling in mature osteoblasts and osteocytes was achieved by overexpression of the GC-inactivating enzyme 11ß-HSD2 under the control of an osteoblast-specific 2.3-kb collagen type Ia1 promotor with a CD1 background (Col2.3-11β-HSD2 tg mice). Mice were generated as originally described by Sher et al. [17] and kindly provided as a gift by Dr. Barbara Kream (Department of Medicine, University of Connecticut Health Center, Farmington, CT, USA). In accordance with Institutional Animal Welfare Guidelines and an approved protocol (2012/006) by the Sydney South West Area Health Services (SSWAHS) Animal Welfare Committee, animals were housed in the animal facility of the ANZAC Research Institute (Sydney, NSW, Australia). For data reporting and storage, we followed the internationally established ARRIVE guidelines [18].

### Initiation and prolongation of K/BxN STIA

Arthritis was induced in 5-week-old Col2.3-11β-HSD2 tg mice (tg) and their wild type (WT) littermates by intraperitoneal (i.p.) injections of 150 μl K/BxN mouse serum on day 0 and day 2. K/BxN mouse serum was obtained from 60-day-old K/BxN mice, who develop spontaneous arthritis due to being crossbred from KRN- and NOD mice. The sera from K/BxN mice were collected, pooled, stored at − 70 °C and injected as previously described [19].

#### STIA

**Fig. 1:**
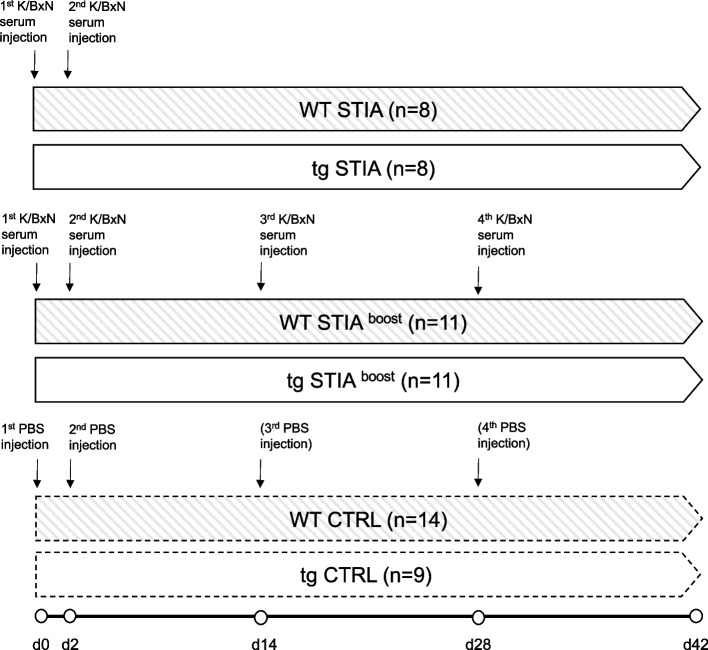
Study design and group distributions according to genotype and arthritis protocol. K/BxN STIA was induced by i.p. injections on days 0 and 2 in WT STIA and tg STIA mice (top row) and boosted with additional i.p. injections on days 14 and 28 in WT STIA ^boost^ and tg STIA ^boost^ animals to maintain a chronic state of arthritis (middle row). CTRL mice were injected with PBS respectively at the same time points (bottom row). As there was no clinical difference between CTRL animals that were injected on days 0 and 2 and those injected on days 0, 2, 14, and 28, CTRL animals were grouped according to their genotype. CTRL, control; i.p., intraperitoneal; PBS, phosphate-buffered saline; STIA, serum transfer-induced arthritis; tg, transgenic; WT, wild type

In a first cohort, the course of arthritis was allowed to naturally abate with the aim of examining the post-acute phase of arthritis and the regenerative processes involved (WT STIA, *n* = 8; tg STIA, *n* = 8). Control (CTRL) animals received 150 μl phosphate-buffered saline (PBS) on days 0 and 2 (WT CTRL, *n* = 5; tg CTRL, *n* = 5) (Fig. 1).

#### STIA ^boost^

To maintain a chronically active prolonged arthritis, a second cohort received repeated i.p. boost injections of 150 μl K/BxN mouse serum on days 14 and 28 (WT STIA ^boost^, *n* = 11; tg STIA ^boost^, *n* = 11). CTRLs received 150 μl PBS at respective time points (WT CTRL, *n* = 9; tg CTRL, *n* = 4). As there were no clinical, histological, or radiological differences observed between CTRL animals who received PBS injections following the STIA or the STIA ^boost^ protocol, the animals were grouped as WT CTRL and tg CTRL. All mice were observed for 42 days (Fig. 1).

### Clinical arthritis assessment

Body weight and clinical parameters of arthritis were assessed daily following arthritis induction on day 0 up to the endpoint on day 42. Ankle joint swelling was assessed daily by measuring the maximum medial to the lateral diameter of ankles using a digital caliper. Measurements from both ankles were averaged for each time point. Since K/BxN STIA typically affects all paws, a scoring system for each limb (2 forelimbs and 2 hind limbs) was applied by two independent and blinded observers (EW and TM) as previously described [11, 20–22]: 0, no swelling; 1, mild-to-moderate swelling and erythematic ankle and/or 1 swollen digit; 2, moderate swelling and erythematic ankle or swelling in 2 or more digits; and 3, marked swelling along all aspects of the paw or all 5 digits swollen. The scores for each paw were added and a maximum score of 12 was possible.

### Tissue collection and specimen preparation

On day 42, animals were euthanized, and samples were harvested for histological and histomorphological assessments as well as for micro-computed tomography (μCT) as previously described [11, 23]. Ankle joints and tibiae were dissected and fixed in paraformaldehyde 4% (PFA)/PBS for μCT analysis (see below). After μCT analysis, ankles and tibiae were decalcified in 10% ethylene diamine tetraacetic acid (EDTA) and embedded in paraffin. Serial sections of 5 μm thickness were obtained to allow a central lateral view of the ankle joint and stained with (1) hematoxylin and eosin (H&E) for an overview of inflammatory changes as well as a (2) toluidine blue (TB) to interpret the degree of cartilage degradation and (3) tartrate-resistant acid phosphatase (TRAP) for identifying and quantifying osteoclast activity and bone resorption [23, 24]. Proximal tibial metaphyses were stained with H&E and TRAP for histomorphometric analyses.

### Histopathological scoring

Histopathological scoring was performed by 2 independent investigators (EW and TM), both blinded to genotype and experimental groups as previously described [11, 23, 24]. Analyses included inflammatory cell infiltration, edema and synovial hyperplasia (H&E), loss of cartilage, chondrocytes and collagen disruption (TB), osteophyte formation, and areas of bone resorption and number of osteoclasts (TRAP).

H&E: Inflammation Score—0, normal; 1, minimal inflammatory infiltration; 2, mild infiltration with no soft tissue edema or synovial lining cell hyperplasia; 3, moderate infiltration with surrounding soft tissue edema and some synovial lining cell hyperplasia; 4, marked infiltration, edema, and synovial lining cell hyperplasia; and 5, severe infiltration with extended soft tissue edema and marked synovial lining cell hyperplasia.

TB: Cartilage Degradation Score—0, normal; 1, loss of toluidine blue staining only; 2, loss of toluidine blue staining and mild cartilage thinning; 3, moderate loss of toluidine blue staining and moderate multifocal cartilage loss; 4, marked loss of toluidine blue staining with marked multifocal cartilage loss; and 5, severe multifocal cartilage loss.

TB: Osteophyte Score [25]—0, none; 1, formation of cartilage-like tissues; 2, increase of cartilaginous matrix; and 3, endochondral ossification.

TRAP: Bone Erosion Score: 0, none; 1, minimal (not readily apparent on low magnification, rare osteoclasts); 2, mild (some areas of resorption not readily apparent on low magnification, some osteoclasts visible); 3, moderate (obvious bone resorption with numerous osteoclasts visible); 4, marked (large erosion areas extending into the bone cortex with numerous osteoclasts visible in all areas); and 5, severe erosions with full-thickness defects in the cortical bone.

### μCT

μCT of tibiae was performed as previously described [11, 23, 26] using a SkyScan 1172 X-ray microtomograph (SkyScan, Bruker microCT, Kontich, Belgium). Scans were performed at 100 kV, 100 μA, and a shutter speed of 590 ms. With a resolution of 6.93 μm/pixel, 1800 X-rays were collected for each scan. For image reconstruction, the GPU Accelerated NRecon software (version 1.6.6.0, SkyScan, Bruker microCT, Kontich, Belgium) was used. Afterwards, samples were analyzed by the CTAn software (version 1.8, SkyScan, Bruker microCT, Kontich, Belgium). Three-dimensional visualization of reconstructed ankles was achieved by using the 3VGStudie MAX 1.2 software (Volume Graphics GmbH, Heidelberg, Germany).

To quantify systemic bone loss, trabecular morphometry of the proximal tibia was assessed, selecting a 1-mm region of interest (ROI) 0.5 mm below the growth plate within the endosteal borders. ROIs defined the measurements of morphological parameters following an automated algorithm in order to determine the bone’s structure, density, and volume: BV/TV, bone volume/total volume (%); Tb.N, trabecular number (/mm); Tb.Th, trabecular thickness (μm); and Tb.Sp, trabecular separation (μm) [11, 23, 26].

For assessment of bone erosions of the cortical, intraarticular bone of the ankle, a semi-quantitative score was applied to both two- and three-dimensional images of the tibiotalar joint: 0, no erosions or cortical damage; 1, mild cortical bone transformation and beginning erosive changes; 2, moderate bone damage comprising erosions and vacuoles; and 3, severe damage with large erosions and full-thickness defects of the cortex. The total score from two independent and blinded observers (EW and TM) was averaged. Of note, radiological bone erosion scoring data was only available for WT and tg CTRL and STIA ^boost^ groups. This explains why there are fewer animals in the CTRL groups.

### Histomorphometry

Histomorphometric analysis was conducted at the proximal tibial metaphysis by using OsteoMeasure (version 1.01, Osteometrics, Inc., Decatur, GA, USA) in conjunction with a light microscope (Leica Microsystems GmbH, Wetzlar, Germany) [11, 23, 26]. A ROI was selected 0.3 mm below the growth plate of the tibia comprising an area of 1.5 × 1 mm. The following histomorphometric parameters were evaluated at a 400-fold magnification: Ob.S/BS, osteoblast surface/bone surface (%); N.Oc/BS, number of osteoclasts/bone surface (/mm); and Oc.S/BS, osteoclast surface/bone surface (%) [11, 23, 26].

### Statistical analysis

To evaluate the course of ankle size, the semi-quantitative arthritis score and body weight, linear mixed-effects models with splines of grade 4 were fitted using the lme4 and lmerTest packages in R. Genotype (WT or tg) and treatment (STIA or STIA ^boost^), both additionally in interaction with time, and binary indicator variables for boosting on day 14 and for boosting on day 28, both set for the entire subsequent observation period, were investigated in the modeling. R package version 4.0.2 was used for analyses.

For remaining secondary endpoint comparisons between groups, the non-parametric two-sided Wilcoxon-Mann–Whitney test was employed. To detect outlier values, the Grubb’s test was performed for each group [27]. Outliers were displayed as square data points and excluded from statistical analyses. Significance was accepted where *p* < 0.05. Endpoint comparisons were conducted, and figures were created using GraphPad Prism 9, version 9.5.0 (GraphPad Software, Boston, MA, USA).

### Sample size calculations

Sample sizes were calculated using the nQuery software. Group sizes of 8 animals with an allocation ratio of 2:1 in favor of STIA compared to CTRL animals were aimed for. This allows to detect large effect sizes of Cohen’s *d* = 2.0 with 80% power and a two-sided type I error rate of 5%, when comparing STIA vs. CTRL with a *t*-test for independent groups. For comparisons between the two STIA groups, effect sizes of *d* = 1.51 could be detected. Yet, the statistical power of the analyses was assumed to be higher, since for each subject, multiple time points of observation (up to 42) were available. Applying appropriate models that account for repeated measurements allowed for larger statistical power, so that smaller effect sizes could also be detected.

## Results

### Long-term effects of experimental arthritis on clinical arthritis, ankle size, and body weight

**Fig. 2:**
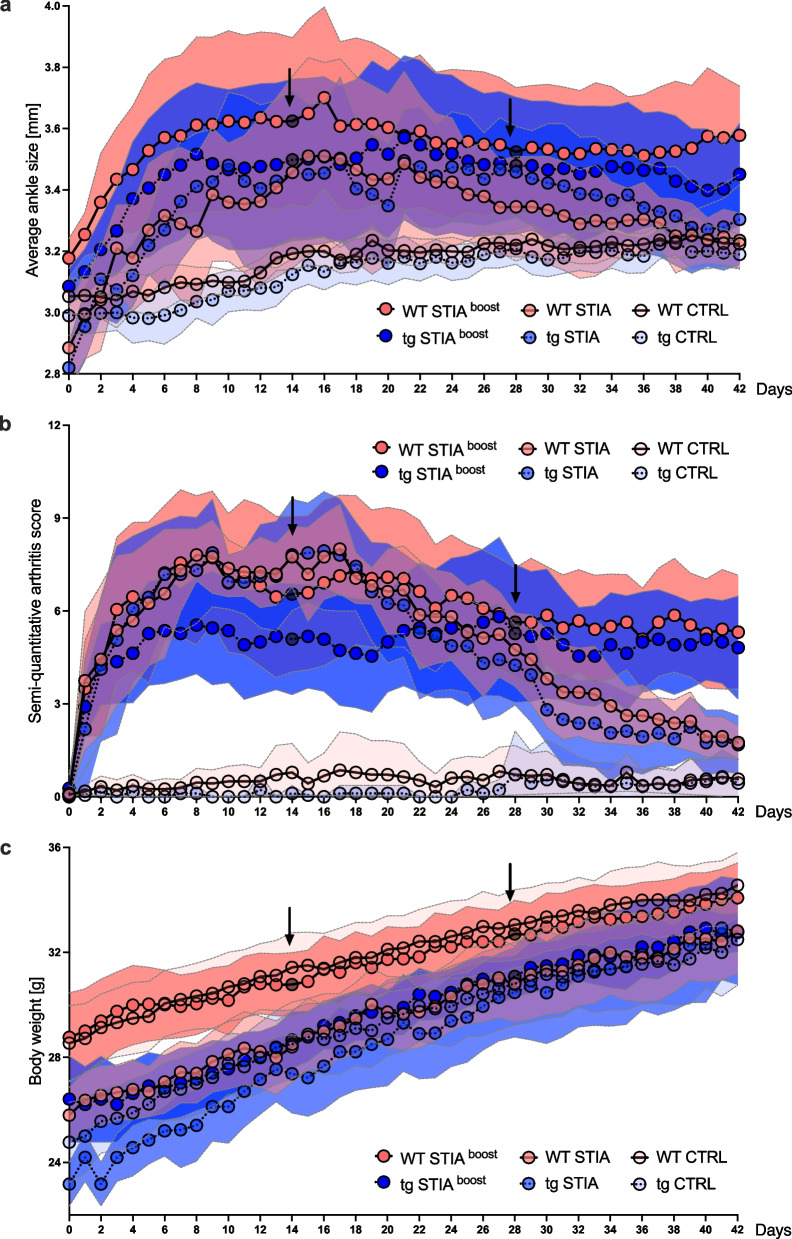
Longitudinal development of **a** ankle size, **b** clinical arthritis score, and **c** body weight over 42 days of STIA for indicated groups. Data points represent means, error bands indicate 95% confidence intervals, and arrows and gray data points boost injections for WT STIA ^boost^ and tg STIA ^boost^ animals on days 14 and 28. CTRL, control; STIA, serum transfer-induced arthritis; tg, transgenic; WT, wild type

Following arthritis induction, WT STIA, tg STIA, WT STIA ^boost^, and tg STIA ^boost^ mice developed an arthritic phenotype, while WT CTRL and tg CTRL animals did not show any signs of arthritis. In all arthritic WT and tg mice, the degree of arthritis increased gradually, peaked around approximately day 8, and plateaued until day 14. In the STIA groups, arthritis then gradually declined until day 42, whereas in the STIA ^boost^ groups, arthritis was maintained at a stable moderate level until day 42, yet only the boost injection on day 28 seemed to influence recurrent ankle swelling and worsening of the clinical arthritis score. Interestingly, we did not detect statistically significant differences between WT and tg animals over time for ankle size measurements (Fig. 2a and Table 1) and only small differences for the clinical arthritis score (Fig. 2b and Table 1).

**Table 1:** Estimates of the linear mixed-effects models for the course of body weight, ankle size, and clinical arthritis score

	Ankle size			Clinical arthritis score			Body weight		
	Estimate	SD	*p*	Estimate	SD	*p*	Estimate	SD	*p*
Intercept	3.03	0.05	< 0.001	0.26	0.42	0.54	28.29	0.48	< 0.001
Day	0.30	0.02	< 0.001	0.89	0.27	0.001	3.78	0.12	< 0.001
Day^2^	0.19	0.02	< 0.001	− 0.87	0.25	0.001	4.85	0.11	< 0.001
Day^3^	0.33	0.04	< 0.001	− 0.33	0.48	0.48	7.91	0.22	< 0.001
Day^4^	0.21	0.02	< 0.001	− 0.75	0.23	0.001	4.82	0.11	< 0.001
Genotype tg	− 0.04	0.06	0.49	− 0.24	0.47	0.61	− 3.08	0.54	< 0.001
Genotype tg * day	0.02	0.02	0.32	− 0.68	0.27	0.012	1.53	0.12	< 0.001
Genotype tg * day^2^	− 0.01	0.02	0.71	0.19	0.27	0.48	1.42	0.12	< 0.001
Genotype tg * day^3^	− 0.08	0.04	0.06	− 0.62	0.52	0.23	2.67	0.24	< 0.001
Genotype tg * day^4^	0.02	0.02	0.29	0.56	0.24	0.023	1.56	0.11	< 0.001
STIA	0.07	0.06	0.24	2.48	0.49	< 0.001	− 0.32	0.55	0.57
STIA * day	0.19	0.02	< 0.001	2.76	0.28	< 0.001	− 0.12	0.13	0.33
STIA * day^2^	0.06	0.02	0.009	− 0.10	0.27	0.72	0.01	0.12	0.96
STIA * day^3^	0.51	0.04	< 0.001	5.81	0.54	< 0.001	− 0.39	0.24	0.11
STIA * day^4^	− 0.18	0.02	< 0.001	− 2.72	0.25	< 0.001	0.59	0.11	< 0.001
Boost day 14	− 0.10	0.01	< 0.001	0.09	0.12	0.44	− 0.49	0.06	< 0.001
Boost day 28	0.03	0.01	0.004	1.50	0.12	< 0.001	− 0.42	0.06	< 0.001

*SD* Standard deviation, *STIA* Serum transfer-induced arthritis

*indicates interactions between variables

Body weight gain was higher in tg than in WT mice, with a lower baseline body weight for tg mice. Regardless of the arthritis protocol (STIA vs. STIA ^boost^), body weight gain was similar within each group of WT and tg mice following disease induction (Fig. 2c and Table 1). Body weight gain flattened in mice receiving the STIA ^boost^ protocol compared to mice receiving the STIA protocol, regardless of genotype. It must be considered that baseline body weight was lower in mice receiving the STIA protocol (Fig. 2c and Table 1). When modeling the course of body weight gain, genotype (WT vs. tg) but not STIA treatment was significant both as a single term and in interaction with time (*p* < 0.001). Also, boosting on day 14 and 28 was recognized as influential in the model fit.

### Intraarticular inflammation and cartilage degradation following long-term arthritis exposure

**Fig. 3:**
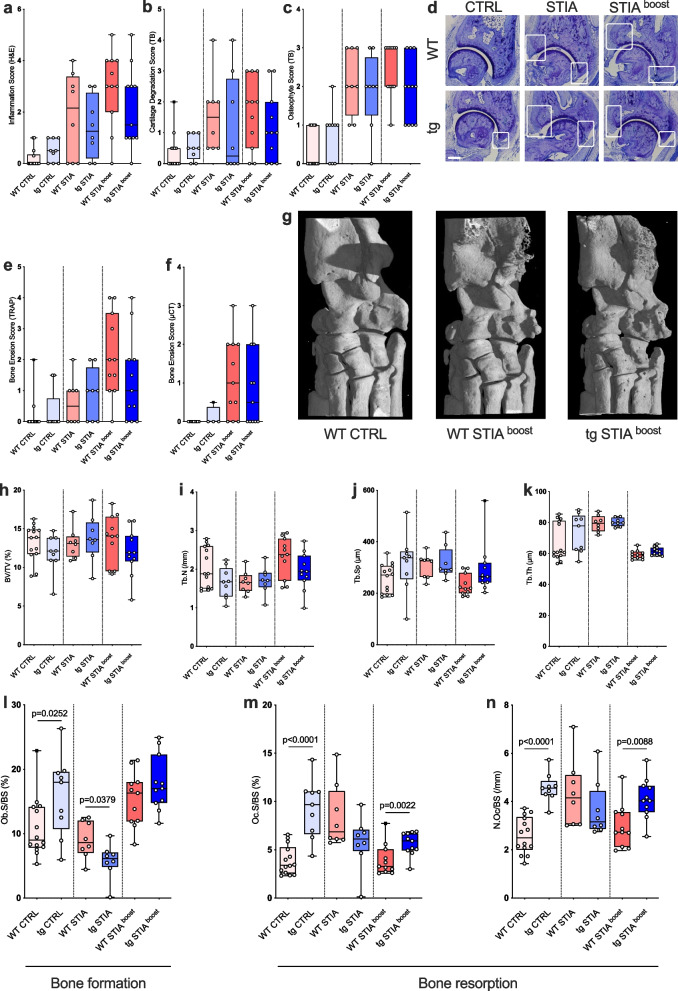
Histopathological scores for **a** intraarticular inflammation (H&E), **b** cartilage degradation (TB), and **c** osteophyte formation (TB) of tibiotalar joints on day 42. **d** Representative histological sections with white boxes capturing osteophyte formation (TB). Scale bar: 200 μm. **e** Histological Bone Erosion Score (TRAP) and **f** μCT-based Bone Erosion Score for ankle joints on day 42. **g** Representative three-dimensional reconstructions of ankles of WT CTRL, WT STIA ^boost^, and tg STIA ^boost^ animals on day 42. Systemic bone changes analyzed by μCT for **h** bone volume (BV/TV), **i** trabecular number (Tb.N), **j** trabecular separation (Tb.Sp), and **k** trabecular thickness (Tb.Th) for indicated groups on day 42. Histomorphometric indices for bone formation, including **l** osteoblast surface (Ob.S/BS), and for bone resorption, including **m** osteoclast surface (Oc.S/BS) and **n** number of osteoclasts (N.Oc/BS) for indicated groups on day 42. Boxplots show median (line), interquartile range (box), and minimum and maximum (whiskers). Black squares indicate outliers. CTRL, control; STIA, serum transfer-induced arthritis; tg, transgenic; WT, wild type

Fourty-two days following arthritis induction, joint inflammation and cartilage degradation were still present in WT and tg animals, yet no significant differences were detected between genotypes for neither STIA nor STIA ^boost^ groups (Fig. 3a, b).

### Effects of long-term arthritis on local osteophyte formation and intra- and periarticular bone erosions

A central question to be addressed by the prolonged arthritis model is that of appositional osteophyte formation and bone erosions in a chronic inflammatory setting. On day 42, osteophytes and bone erosions were assessed at the arthritic site of the tibiotalar joint by histology and μCT. Similar to histopathological findings for inflammation, the histopathological Osteophyte Score was significantly higher for WT (STIA, *p* < 0.0001; STIA ^boost^, *p* < 0.0001) and tg (STIA, *p* = 0.0170; STIA ^boost^, *p* = 0.0067) mice compared to CTRLs (Fig. 3c, d). The Bone Erosion Score was also significantly higher for WT (STIA, *p* = 0.0117; STIA ^boost^, *p* < 0.0001) and tg STIA ^boost^ (*p* = 0.040) animals compared to CTRLs, while there was no difference between genotypes for both histopathological scores (Fig. 3e).

For the analysis of local bone erosions by μCT, three-dimensional reconstructions of the ankles were scored by a semi-quantitative bone erosion score (Fig. 3f). When compared to CTRLs, bone erosions were evident in WT STIA ^boost^ and tg STIA ^boost^ animals (Fig. 3g), yet only reaching statistical significance for WT mice (*p* = 0.002). No significant difference was found between genotypes (Fig. 3f).

### Systemic effects of long-term arthritis on bone morphology

Furthermore, μCT analyses of proximal tibiae were performed to quantify the systemic effects of inflammation on bone morphology in a long-term setting.

No significant differences were observed for bone volume (BV/TV, Fig. 3h), trabecular number (Tb.N, Fig. 3i), and trabecular separation (Tb.Sp, Fig. 3j). Compared to CTRLs, trabecular thickness (Tb.Th) was significantly decreased in tg STIA ^boost^ compared to CTRL animals (*p* = 0.0381) without any differences between genotypes (Fig. 3k).

To elucidate whether changes in systemic bone morphology observed in μCT were due to high or low bone turnover, histomorphometric analyses were performed at the proximal tibia.

Osteoblast surface (Ob.S/BS) was significantly larger in WT STIA ^boost^ (*p* = 0.0031) and smaller in tg STIA (*p* = 0.0016) animals compared to CTRLs. Further significant differences were observed between WT CTRL and tg CTRL (*p* = 0.0252) animals where tg animals showed greater osteoblast surface areas and between WT STIA and tg STIA animals (*p* = 0.0379) where WT animals showed higher values (Fig. 3l).

Coherently, osteoclast surface (Oc.S/BS) was also significantly larger in WT STIA (*p* = 0.0006) and smaller in tg STIA (*p* = 0.0360) animals compared to CTRLs (Fig. 3m).

When comparing CTRL groups, higher values were further seen for both bone resorption parameters (osteoclast surface (Oc.S/BS) (Fig. 3m) and number of osteoclasts (N.Oc/BS) (Fig. 3n)) in tg compared to WT mice (both *p* < 0.0001). Last, tg STIA ^boost^ animals displayed higher values compared to WT STIA ^boost^ animals for the same indices: Oc.S/BS, *p* = 0.0022 (Fig. 3m); N.Oc/BS, *p* = 0.0088 (Fig. 3n).

## Discussion

In the originally described study by Buttgereit et al. from 2009, K/BxN STIA and joint destruction were significantly attenuated by tg disruption of GC signaling in osteoblasts [11]. While these findings were highly significant for ankle size and the clinical arthritis score as well as for histopathological subscores of inflammation and cartilage degradation, they were less conclusive for bone erosion indices on day 14. Since the original experiments were conducted over a period of two weeks only, and bone erosions and systemic inflammatory bone loss, at least in theory, might take longer to evolve, we decided to prolong the course of arthritis using a long-term model with repeated boost injections of K/BxN autoantibodies on days 14 and 28. In a second experiment, we investigated the effects of abrogated endogenous GC signaling in osteoblasts and osteocytes on the underlying resolution process of arthritis by allowing arthritis to gradually abate by its natural transient course until day 42.

We were able to show that by repeatedly injecting K/BxN serum i.p., the course of arthritis was effectively prolonged. However, no significant difference between WT and tg mice was observed, regardless of the arthritis protocol (STIA and STIA ^boost^), for the clinical course of arthritis over 42 days.

Correspondingly, no significant differences were observed between genotypes for histological indices of inflammation, cartilage degradation, osteophyte formation, and bone loss on day 42. It appears that the effect of abrogated GC signaling in osteoblasts on overall inflammation and subsequent structural alterations has less impact over time.

Similar results were previously reported by us for endpoint comparisons between WT and mutant mice on day 48 following antibody-mediated arthritis. While significant differences in histological scores for inflammation were present on day 10, these could not be seen on day 48, underlining the limitations of transient autoantibody-induced arthritis models in mimicking a chronic disease state [21, 22].

It was previously discussed that endogenous GCs may worsen inflammation and corresponding intraarticular damage through immunostimulatory patterns when present at low tissue concentrations [28, 29]. Although this was the case in the current study, we did not observe differences between genotypes after 42 days of inflammation. As the original work by Buttgereit et al. showed significantly less intraarticular inflammation and cartilage damage in tg compared to WT animals on day 14 following arthritis induction [11], this effect may disappear over time and only be attributed to acute and subacute arthritis.

Although not statistically significant, histomorphometry results for WT animals were largely similar to those described by Buttgereit et al. [11]. Bone formation (Ob.S/BS) was lower, and bone resorption (Oc.S/BS and N.Oc/BS) was higher in WT STIA animals when compared to CTRLs. This was expected, as arthritis-induced bone loss is caused by a disrupted coupling between bone formation and resorption [30, 31]. In RA, the existing dysbalance in bone homeostasis is not only a result of inflammation-induced osteoclastogenesis, but also a result of an impaired capacity of osteoblast-lineage cells to form bone [32] resulting in a net bone loss. This is likely to be due to the inhibition of Wnt signaling by Dickkopf-related protein (DKK)-1 and secreted Frizzled-related protein (sFRP) families of Wnt signaling antagonists [33].

We further observed increased signs of bone turnover in tg mice for both, the CTRL and the STIA ^boost^ group. In previous experiments, we have observed that tg mice seem to have an intrinsically higher bone turnover compared to WT animals which is particularly reflected in bone formation and resorption markers [6, 10]. Interactions of endogenous GCs with Wnt signaling have been debated, yet clear mechanistic explanations are currently not available [6, 10].

Interestingly, we saw a reversed regulation in animals that received the STIA protocol. The long-term STIA protocol allows arthritis to naturally abate, which leaves animals in a recovery state after 42 days. This state between health and inflammation may explain the reversed results for bone turnover markers.

Although histomorphometric indices were previously reported to be similar in healthy and arthritic WT animals overexpressing osteoblastic and osteoclastic 11ß-HSD2 [34, 35], a trend similar to our current results was previously described for the CAIA model [12]. There is further evidence of endogenous GCs to impair bone quality in aging mice, although our mice were relatively young compared to the reported study animals by Weinstein et al. [36]. Whether the observed effects are mainly driven by the arthritis model or the transgenic genotype remains speculative.

We conclude the effect of GC signaling in osteoblasts on arthritis to be of minor importance in a long-term setting. Bearing in mind the various cytokines and primary inflammatory cells that are involved in orchestrating autoimmune processes, osteoblasts are unlikely to call the tune. Rather pathologically altered structures that are a source of potent drivers of inflammation, such as the synovium and subchondral bone, are likely to dominate the course of inflammation in autoimmune arthritis, as laid out in a summary by Mathiessen et al. [37].

When regarding the dwindling differences between tg and WT arthritic mice over time, another factor that could have influenced the results is the genetic status of tg mice. If expression of the transgene was impaired, a reduced effect in the modulation of arthritis could have occurred. In general, a certain degree of instability for tg models exists, with the expression being prone to decrease over time and with several breeds [38].

Matzelle et al. showed that in the long-term K/BxN STIA model, bone formation was significantly increased, due to changes in Wnt signaling [16]. In accordance, we detected an increased osteophyte formation during long-term K/BxN STIA in both genotypes which may counterbalance the apparent systemic bone loss and local bone erosions.

This study has some limitations. Although the K/BxN STIA model exhibits similarities to human RA, its induction through preformed autoantibodies is mostly independent of T- and B-cells, which are highly relevant in human inflammatory joint diseases [39]. Moreover, while the murine K/BxN model affects the joints symmetrically similar to human RA, its transient nature differs from the chronic disease progression in affected patients. To address this, we chose the STIA ^boost^ protocol, mimicking rheumatic flares, next to the conventional STIA protocol. When comparing our data to the study by Buttgereit et al. [11], differences in animal numbers between the studies may have also had an effect on the reported results, although sample size calculations were followed. To minimize variations in the arthritic potency of the K/BxN mouse serum, sera from K/BxN mice were pooled, before being injected into experimental mice. It cannot be ruled out that the arthritic potency of the K/BxN serum used in our study was not as high as in the original study. Baseline body weight differences between genotypes may have also influenced the disease course, although these differences were also previously described [11]. As we did not detect significant differences in clinical parameters (clinical arthritis score and ankle size), we did not collect and analyze blood samples. In future studies, serum samples could provide information on circulating protein patterns following long-term arthritis exposure. Last, genetic variations in mutant mice are possible, but unlikely in this context, since thorough and standardized genotyping was conducted repetitively.

## Conclusions

To summarize, this study advances our knowledge of the GC-dependent role of osteoblasts in the modulation of inflammatory arthritis. In the chronically persistent inflammation as well as in the resolution phase of K/BxN STIA, disrupted GC signaling in osteoblasts seems to only have a minor impact on inflammatory and reparatory processes. These results narrow the role of endogenous GC signaling by osteoblasts down to early inflammatory processes.

## Data Availability

All relevant data are reported in the manuscript. The raw datasets used and analyzed during the current study are available from the corresponding author upon reasonable request.

## Abbreviations

11ß-HSD2: 11Beta-hydroxysteroid dehydrogenase type 2
AIA: Antigen-induced arthritis
BV/TV: Bone volume/total volume
CAIA: Collagen II antibody-induced arthritis
CTRL: Control
DKK-1: Dickkopf-related protein-1
EDTA: Ethylene diamine tetraacetic acid
GC: Glucocorticoid
H&E: Hematoxylin and eosin
i.p.: Intraperitoneal
μCT: Micro-computed tomography
N.Oc/BS: Number of osteoclasts/bone surface
Ob.S/BS: Osteoblast surface/bone surface
Oc.S/BS: Osteoclast surface/bone surface
OPG: Osteoprotegerin
PBS: Phosphate-buffered saline
RA: Rheumatoid arthritis
ROI: Region of interest
sFRP: Secreted frizzled-related protein
SSWAHS: Sydney South West Area Health Services
TB: Toluidine blue
Tb.N: Trabecular number
Tb.Sp: Trabecular separation
Tb.Th: Trabecular thickness
TRAP: Tartrate-resistant acid phosphatase
tg: Transgene
WT: Wild type
